# Chest CT examinations in patients presenting with acute chest pain: a pictorial review

**DOI:** 10.1007/s13244-015-0429-6

**Published:** 2015-09-15

**Authors:** Sebastiaan Hammer, Lucia J. Kroft, Alberto L. Hidalgo, Ruben Leta, Albert de Roos

**Affiliations:** Department of Radiology, C2-S, Leiden University Medical Centre, Albinusdreef 2a, 2333 ZA Leiden, The Netherlands; Cardiac Imaging Unit, Hospital de la Santa Creu i Sant Pau, Clínica Creu Blanca, Universitat Autònoma de Barcelona, Reina Elisenda de Montcada 17, Barcelona, 08034 Spain; Cardiology Department, Hospital de la Santa Creu i Sant Pau, Clínica Creu Blanca, Universitat Autònoma de Barcelona, Reina Elisenda de Montcada 17, Barcelona, 08034 Spain

**Keywords:** Acute chest syndrome, Chest pain, Chest computed tomography, Cardiac imaging techniques, Emergency care

## Abstract

Acute chest pain (ACP) is one of the most common presenting symptoms at the emergency department. The differential diagnosis is vast. To exclude life-threatening causes, radiologists encounter an increasing amount of thoracic computed tomography (CT) examinations including CT angiography of the heart and great vessels. The dual- and triple-rule CT examinations are currently implemented in clinical practice. We retrospectively identified chest CT examinations in the setting of acute chest pain in our hospitals and collected a variety of common and uncommon cases. In this pictorial essay, we present the most educative cases from patients who presented with acute chest pain in the emergency department of our hospitals and for whom a thoracic CT was ordered. When aortic emergencies, acute coronary syndrome, and pulmonary embolism are excluded, these cases may help the radiologist to suggest alternative diagnoses in the diagnostic challenge of acute chest pain.

*Teaching Points*

• *The number of chest CT examinations for ACP is increasing*.

• *Chest CT examinations may help suggesting alternative diagnosis in ACP*.

• *Radiologists should be aware of the differential diagnosis of ACP*.

## Introduction

Acute chest pain is one of the most common presenting symptoms at the emergency department (ED), up to 10 % of all ED admissions in the United States [1]. The differential diagnosis is vast and includes aortic, coronary artery, pericardial and pulmonary diseases such as pulmonary embolism, thereby frequently providing a diagnostic challenge. Although the primary diagnostic goal is to exclude life threatening causes such as acute coronary artery syndrome (ACS, referred to as a symptomatic coronary artery stenosis or myocardial infarction, i.e., reduced coronary artery flow leading to acute cardiac ischemic states), in only a minority of the patients with acute chest pain ACS is there ultimately a confirmed diagnosis, yielding a substantial number of possible alternative diagnosis. On the contrary, missing the diagnosis of ACS accounts for frequent litigation and is reported in patients with atypical presentations, such as those of younger age or without electrocardiography (ECG) abnormalities [2]. In the last decade, computed tomography (CT) imaging has become a powerful tool in the diagnostic process, aiming to decrease false negative outcomes and providing clues for an alternative diagnosis.

Different modalities have been proposed in the workup of suspected ACS. In the (sub) acute setting the availability and speed of ECG gated CT has lead mainly to an increase in acute thoracic CT exams. A variety of scanning protocols have been developed and extensively studied for this diagnostic question. The expected increase in chest “rule-out” CT scans in patients with acute chest pain stresses the need for radiologists to be aware of an alternative diagnosis. We retrospectively identified patients who presented with acute chest pain at the ED in our hospitals, and selected the most educative cases, not aiming to present a complete overview of all causes of chest pain. The present pictorial essay reviews, thus, some of the alternative diagnoses, focused on aortic, coronary artery, pericardial and pulmonary diseases, which may help the general radiologist in clinical practice.

## Aortic disease

### Aortic aneurysm

**Fig. 1:**
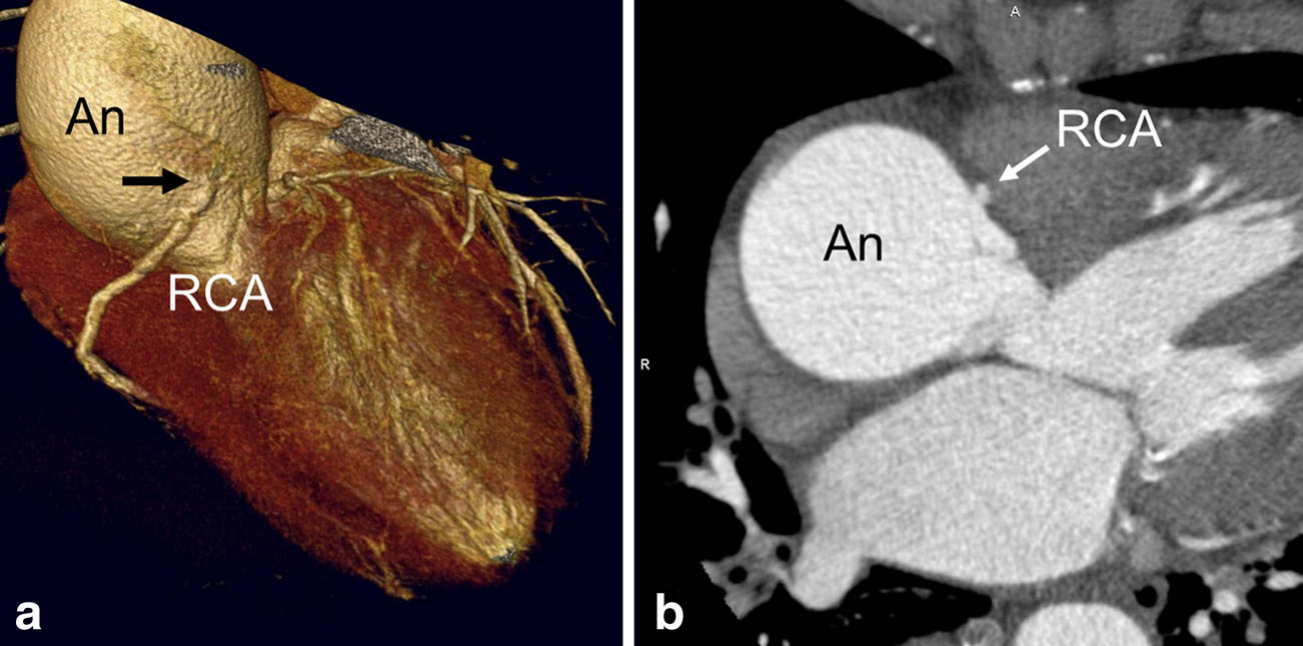
A 59-year female patient with a history of exertional chest pain and dyspnoea presented with progressive chest pain at night. ECG showed ST elevations. Invasive coronary angiography was attempted to detect coronary artery disease under suspicion of acute myocardial infarction. The procedure was technically unsuccessful because of a large aneurysm of the aortic root that hampered selective catheterization of the right coronary artery (RCA). Therefore, chest CT angiography (CTA) was performed. (**a,b**) ECG-gated cardiac CTA, (**a**) volume rendering and (**b**) MIP image, shows a large ascending aorta aneurysm (An) with displacement of the RCA that resulted in an acute angle at the origin of the RCA (black arrow in **a**) that caused RCA ostial-stenosis. Patient had atherosclerotic plaques with approximately 50 % stenosis in the left anterior descending and circumflex coronary artery as well (not shown)

Aortic emergencies have been recently reviewed [3]. Complementary, incidentally found, aneurysms of the thoracic aorta are frequently encountered and can be associated with acute chest pain as well. The upper limit of the aortic diameter is dependent on patient sex, age, and body size. Aortic aneurysms may directly compromise a coronary artery and cause acute chest pain. The causes of aortic dilatation include loss of elastic fibers due to aging (degenerative dilatation, as in the present case), or genetic causes such as Marfan syndrome, Ehlers-Danlos, bicuspid aortic valve disease, or Loeys Dietz syndrome. The normal growth rate of the ascending aorta is approximately 0.07 to 0.2 cm per year, although this expansion may be accelerated by increasing aortic size, in hypertensive patients and in patients with connective tissue diseases [4] Fig. 1.

### Aortic aneurysm; sinus of valsalva aneurysm

**Fig. 2:**
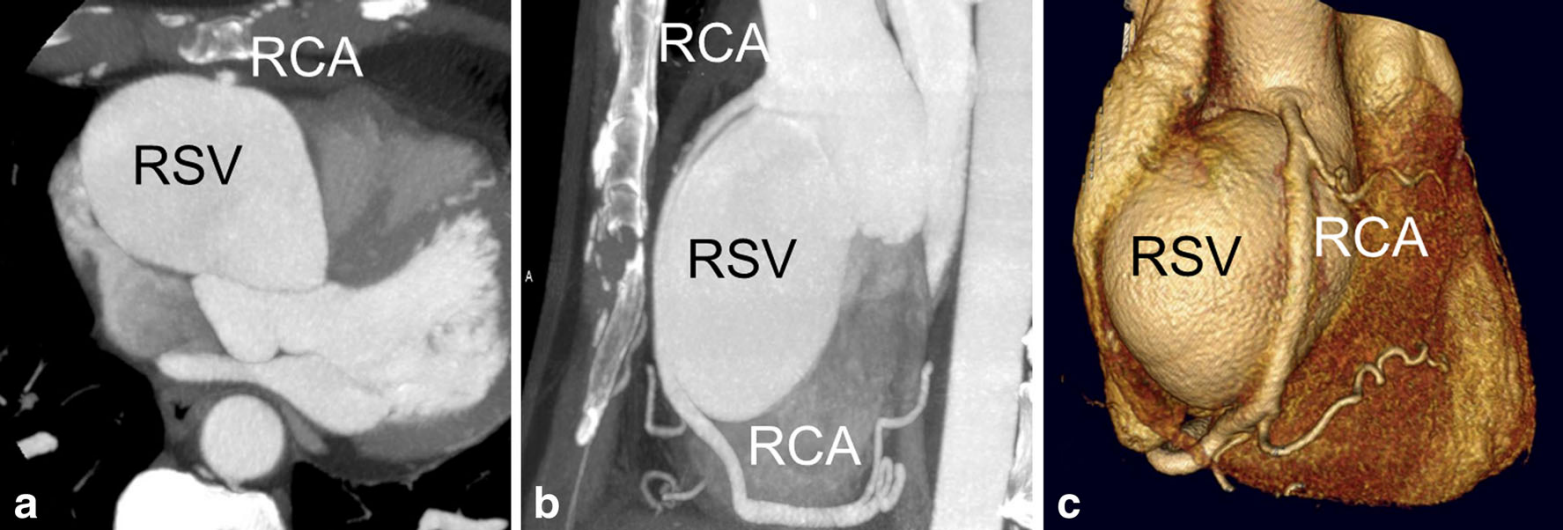
A 53-year male patient with history of pulmonary artery stenosis, presented with exertional chest pain. ECG showed ST segment depressions and left ventricular hypertrophy. At invasive coronary angiography, it was not possible to selectively catheterize the right coronary artery. An aneurysm was observed but the exact location remained unclear. Patient underwent coronary CTA for locating the aneurysm and imaging the right coronary artery. (**a-c**) CTA with maximum intensity and volume rendering reconstructions shows 5.9 × 5.2 × 5.2 cm diameter aneurysm of the right sinus of Valsalva (RSV). (**a**) axial image showing relation with the normal aortic root. (**a-c**) The RCA was stretched out over the RSV aneurysm that caused some narrowing of the RCA lumen. No atherosclerotic coronary artery stenoses were found

Most sinus of Valsalva aneurysms are congenital and associated with other congenital cardiac anomalies, accounting for only 0.1-3.5 % of all congenital heart defects [5]. Although these aneurysms may be found incidentally [6], some may present with angina pectoris by compromising the lumen of the right coronary artery [7], or may present as acute coronary syndrome after rupture, most often in the right ventricle leading to a large shunt [5] [8]. Pericardial effusion may be observed as well, Fig. 2.

### Aortitis

**Fig. 3:**
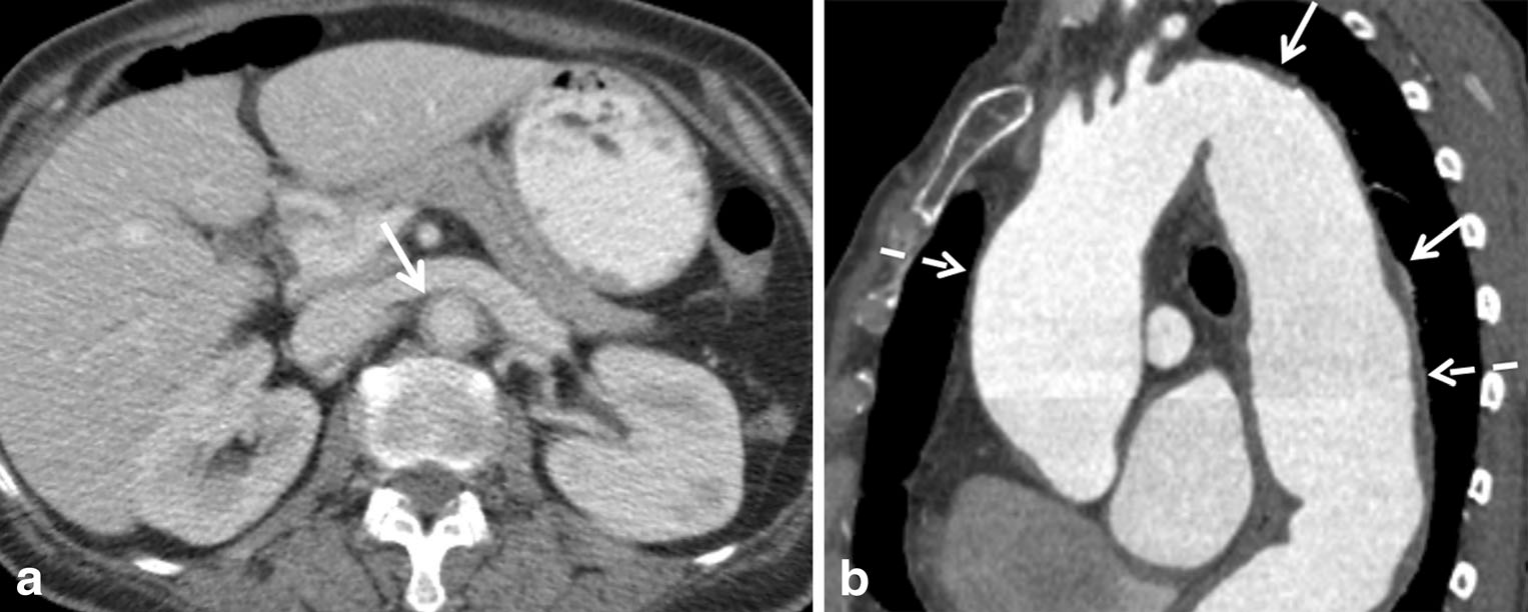
A 58-year-old woman presented with intermittent episodes of atypical, acute chest pain/ epigastric pain and night sweats. In an earlier episode (elsewhere) she had received gastroscopy and a CT scan of the abdomen that showed no abnormalities. At our hospital, blood was drawn that showed anaemia with a haemoglobin level of 5.1 mmol/L. A CT was performed under suspicion of malignancy. (**a**) Contrast enhanced axial CT shows a thickened aortic wall (arrow), consistent with large vessel vasculitis. Biopsy of the temporal artery proved temporal arteritis, consistent with the diagnosis of giant-cell arteritis. Pericardial effusion was also present (not shown). (**b**) Sagittal curved reformat of a contrast enhanced CT in a patient with known Takayasu arteritis, showing wall thickening (solid arrows) and an aneurysm of the ascending and descending aorta (dashed arrows)

Aortitis is a non-specific term, defined as inflammatory infiltration of the aortic wall. The causes are divided into infectious and non-infectious [9]. The most common causes of aortitis are noninfectious etiologies such as Takayasu’s and Giant cell arteritis although other rheumatologic related diseases may be implicated. Aortitis is a relatively uncommon disease entity, but the non-specific clinical presentation (including chest pain) should raise early suspicion. Imaging aims to review the extent of the disease, wall abnormalities, and luminal changes [10]. Moreover, aortitis is associated with an increased risk for acute aortic syndrome including aortic dissection. For diagnosing aortitis, CT is a well-accepted first modality of choice and shows irregular thickening of the aortic wall, which may enhance focal dilatation or stenosis. Moreover, periaortic fat stranding can be seen. Fluid collections, soft tissue mass, and extensive fat stranding may indicate infectious aortitis Fig. 3.

## Coronary artery disease

### Coronary anomaly

**Fig. 4:**
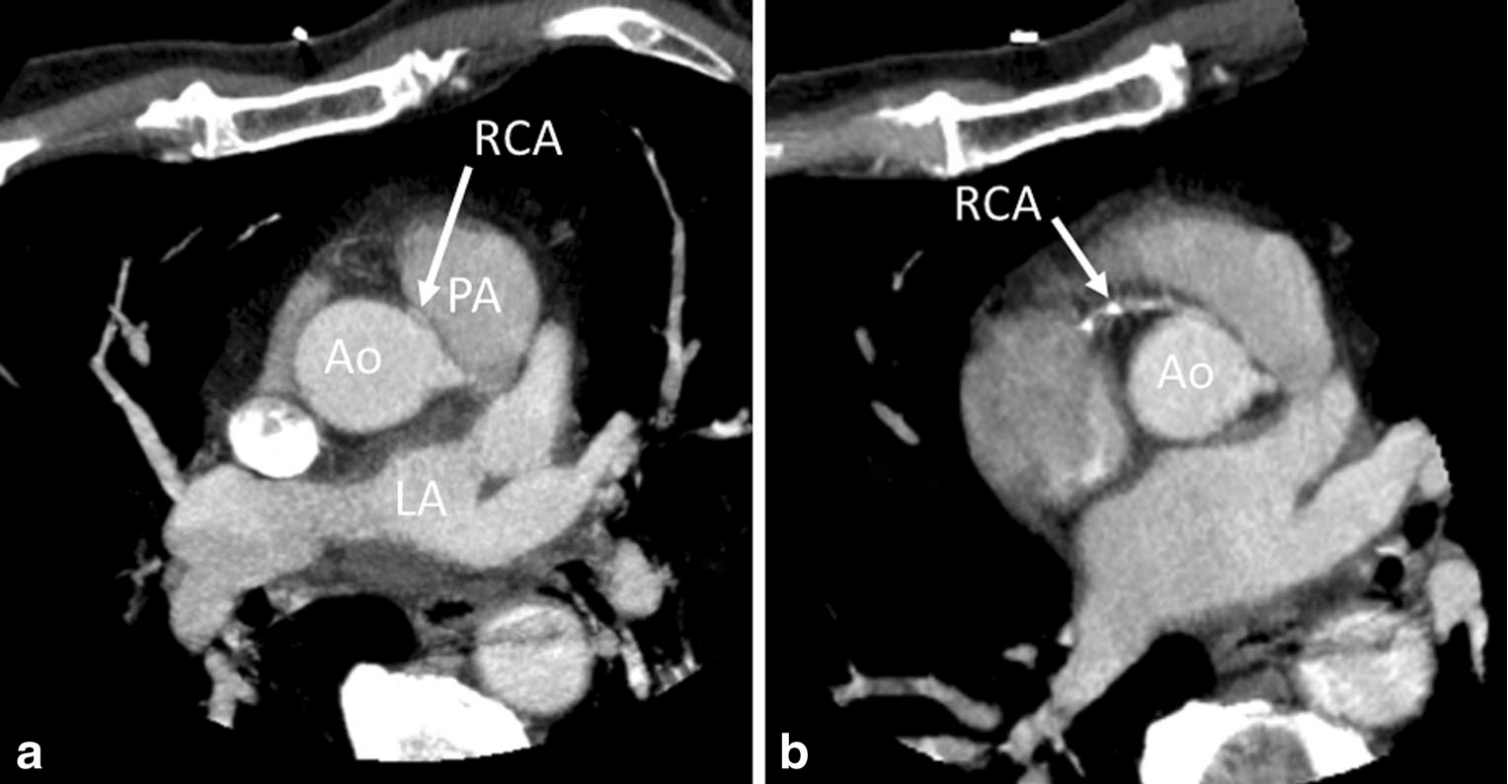
A 73-year male patient presented with acute chest pain. His history revealed extensive coronary artery disease diagnosed as invasive angiography because of angina, although it was impossible to selectively image the right coronary artery (RCA), possibly because of an anomalous origin. Conventional angiography was repeated in our hospital, to visualize the aberrant RCA. During the procedure, patient had asystoly and resuscitation. Emergency coronary CTA was performed, after which the patient underwent emergency bypass surgery. (**a,b**) Coronary CTA in oblique transverse view of the aortic root. Aberrant RCA running between the aorta (Ao) and pulmonary artery (PA, malignant variant), arrow in **a** and **b**. The RCA was of poor quality with multiple stenosis (arrow in **b**). Note the narrow origin and proximal part of the RCA arising near the left coronary cusp at approximately 2 o’clock with a sharp angle with the aorta (normal origin is at right coronary cusp at approximately 11 o’clock, arising with perpendicular orientation from the aorta). Significant three-vessel disease was present. The patient underwent emergency bypass surgery. LA, left atrium

Coronary anomalies are relatively rare (0.3-5.6 %) and are usually classified as benign or malignant [11]. The anomalous interarterial course of a coronary artery, i.e., course between the aorta and pulmonary artery at the level of the right ventricular outflow tract is considered a malignant variant, although typically they are found incidentally and are, thus, asymptomatic. The course of the vessel, however, may result in compression and ischemia, especially during exercise when the aortic and pulmonary root distends. The most common variant is an anomalous RCA originating from the left coronary cusp, in which patients with a high interarterial course are more prone to present with chest pain [12]. A single coronary artery, when coursing interarterially, or the intramural course of a proximal coronary artery through the aortic wall is a malignant variant as well, leading to ACS via the same mechanism. A rare variant that also may lead to ACS is ostial atresia of the left main artery. The left coronary circulation is then supplied with blood from collaterals via the right coronary artery, Fig. 4.

### Coronary artery fistula

**Fig. 5:**
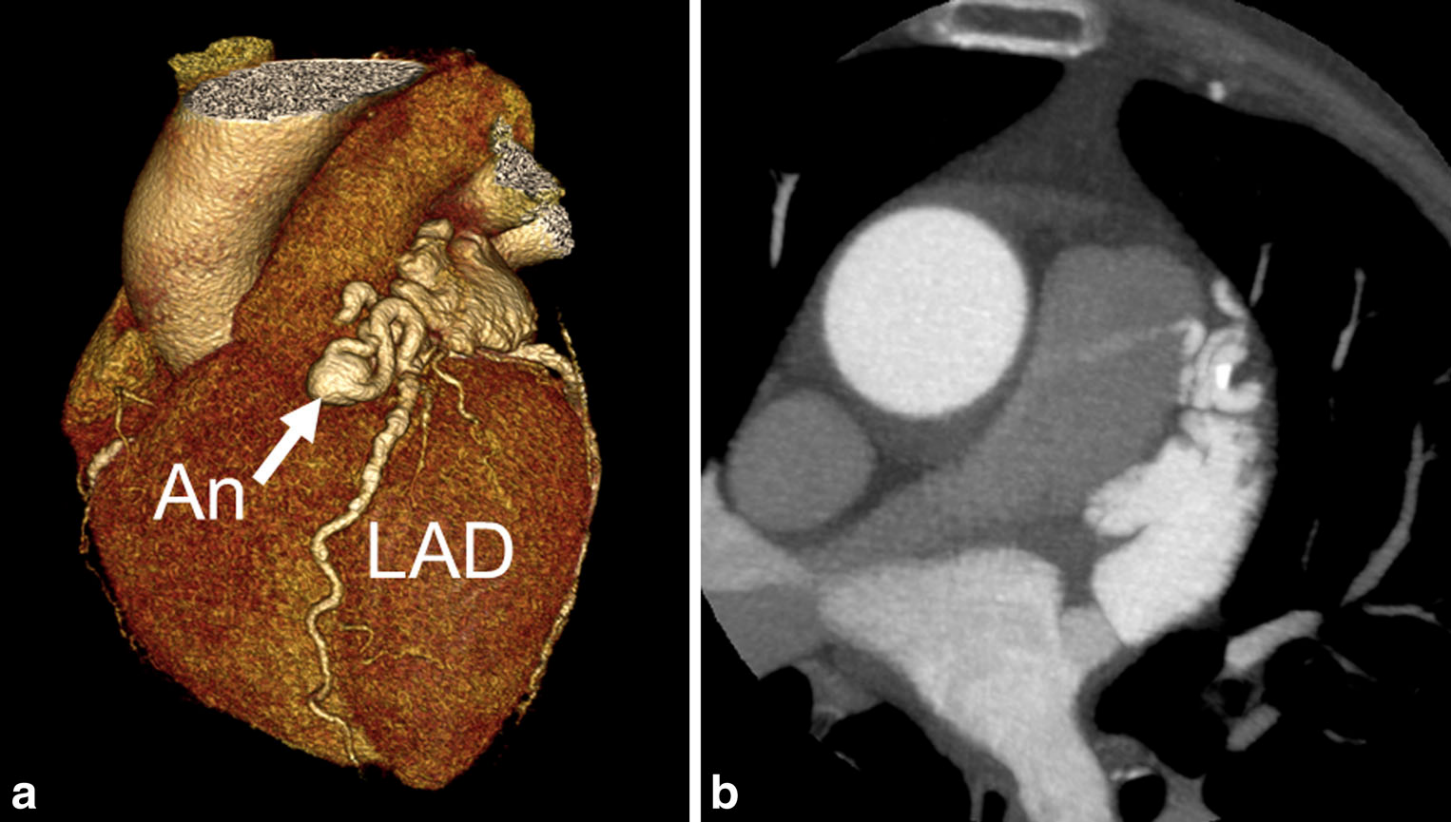
A 75-year female patient with a history of hypertension and type 2 diabetes mellitus presented with atypical complaints resulting in syncope. Echocardiography was suggestive for a coronary artery fistula to the pulmonary artery. (**a**,**b**) Four-mm diameter fistula, (**a**) volume rendering and (**b**) MIP image, arising from the proximal part of the left anterior coronary artery (LAD), with a thirteen-mm diameter aneurysm (An) within the fistula. (**b**) The fistula ends at the main pulmonary artery, note the small contrast jet from the fistula into the pulmonary artery (arrow). Patient had atherosclerotic plaques in the coronary arteries without significant stenosis

Coronary artery fistulas (CAF) form a group of congenital abnormalities characterised by an abnormal connection with a cardiac chamber or great vessel. CAF is encountered in approximately 0.3 %-1.3 % of patients undergoing diagnostic coronary angiography [13]. Although most CAF represent isolated communications, multiple fistulas have been described. Most CAF present as an incidental finding. Nevertheless, depending on the degree of left-to-right shunting, right ventricular dysfunction may be present. Significant shunting may lead to exertional dyspnoea and chest pain, especially during physical activity. The diagnosis of CAF has been traditionally made by coronary angiography [14]. However, since the distal part of the fistula drains into low-pressure chambers or vessels, CAF can be difficult to diagnose at coronary angiography. Cardiac CT better delineates the course of the tortuous vessel and can possibly show a jet into the draining cardiac chamber or vessel. Normal origins of the coronary arteries are seen and feeding vessels may also arise from bronchial or mediastinal arteries, Fig. 5.

### Coronary artery vasospasm

**Fig. 6:**
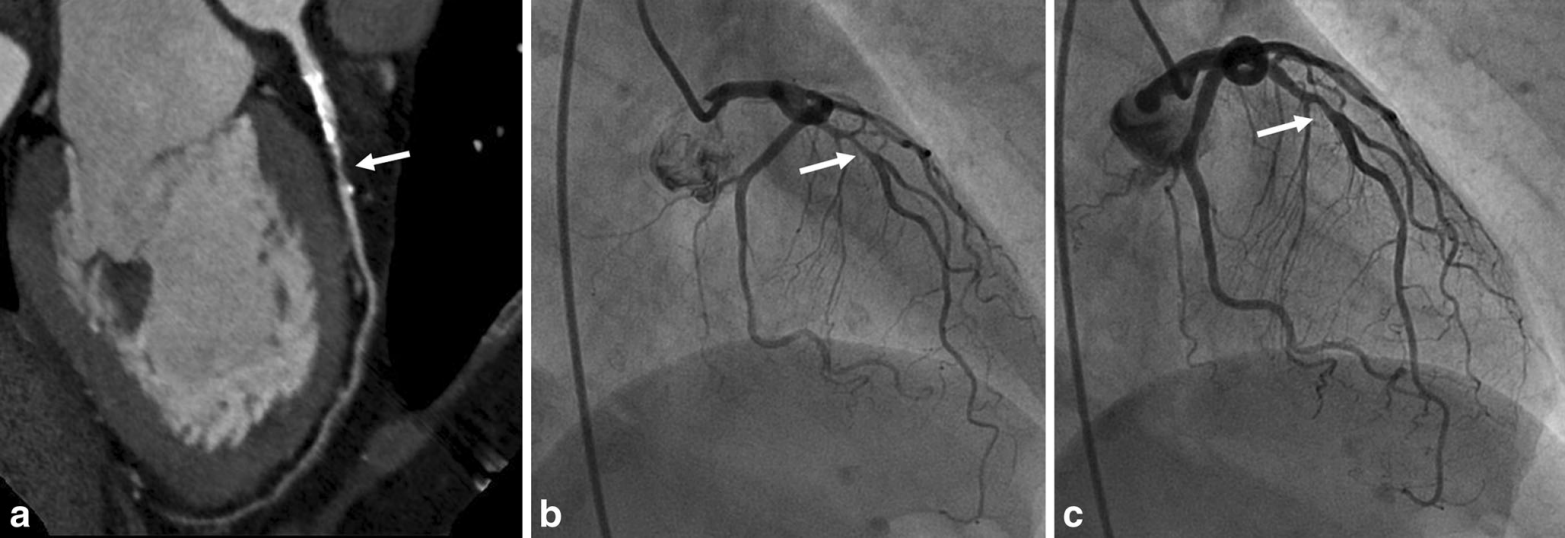
A 48-year female was admitted to the ED after severe chest pain and palpitations. ECG showed transient 1-2 mm ST elevation. Cardiac biomarkers were normal. Cardiac CT was ordered for evaluation of the coronary arteries. (**a**) Cardiac CT performed shows a significant stenosis in the proximal LAD (arrow). (**b**) Invasive coronary angiography shows the same significant lesion. (**c**) After intracoronary administration of nitroglycerin, the lesion became non-significant. The diagnosis of coronary artery vasospasm was made, and interventional procedure was not performed

Coronary artery vasospasm is an important cause of chest pain that can lead to myocardial infarction, ventricular arrhythmias, and sudden death [15]. Chest pain episodes usually occur during the early morning hours. Coronary invasive angiography may or may not demonstrate fixed coronary obstruction. Often an atherosclerotic plaque at the level of vasospasm is present, and; therefore, the detection of a stenosis using cardiac CT may be crucial in interpreting the findings at invasive coronary angiography. The pathophysiology of coronary artery spasm includes endothelial dysfunction through metabolism of nitric oxide [16]. Calcium antagonists and nitroglycerin are effective in treating and preventing new episodes of coronary spasm, Fig. 6.

## Pericardial disease

### Pericarditis

**Fig. 7:**
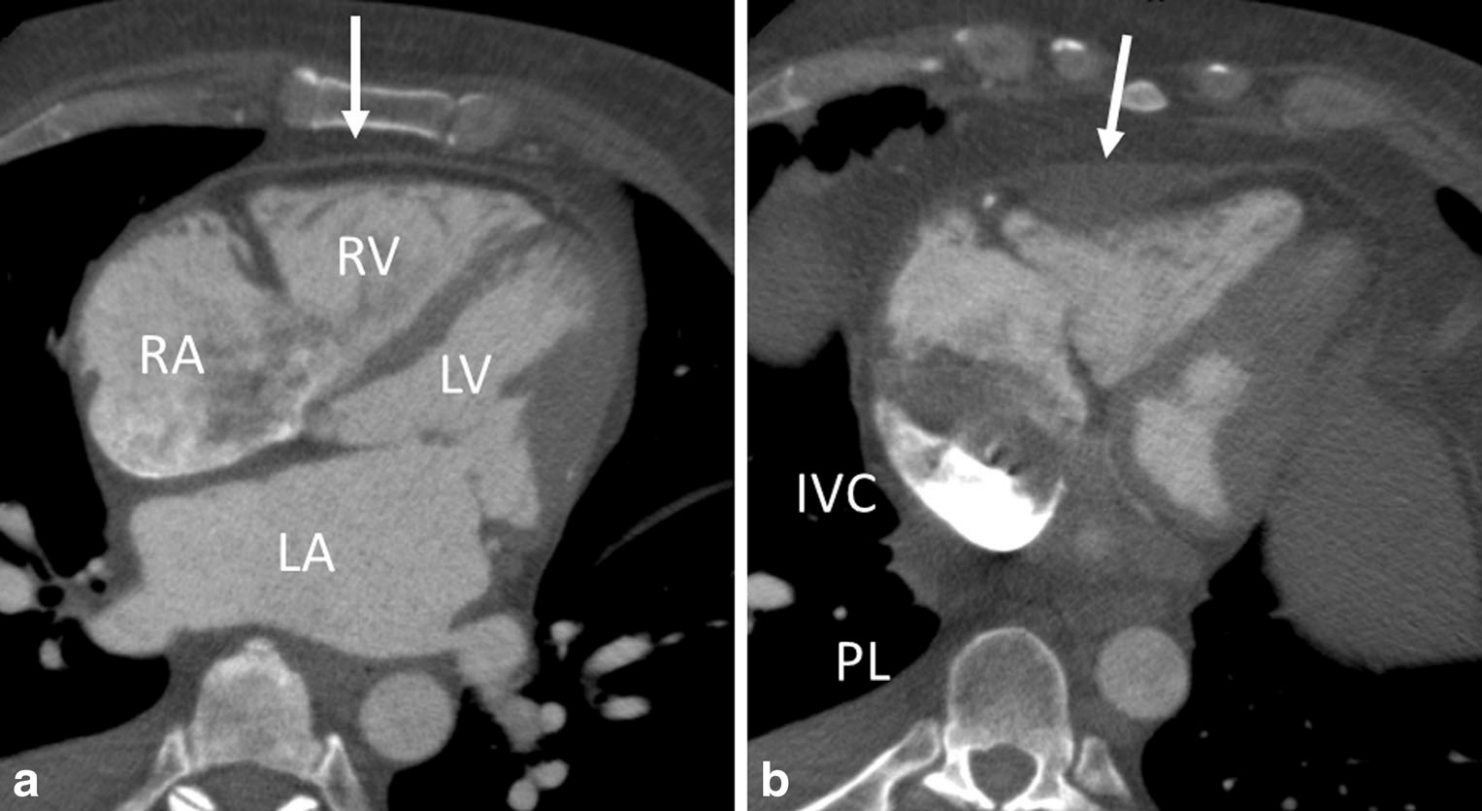
44-year-old male patient with constrictive pericarditis. (**a-b**) CT images in axial views. The pericardium is thickened (arrows). (**a**) The right atrium (RA) and left atrium (LA) are enlarged. (**b**) Note the abnormal shape of the right ventricle (RV) and left ventricle (LV), and inferior vena cava enlargement (IVC). Patient had pleural effusion (PL) as a sign of decompensation cordis. These are among signs of constriction

Pericarditis is a common disorder, and the most common pericardial disease. It typically presents with sharp pleuritic chest pain due to irritation of the pericardium. Any type of pericardial effusion (inflammatory or hemorrhagic) may eventually result in constrictive pericarditis. Causes include post myocardial infarction (Dressler syndrome), post-surgical, after irradiation therapy, infectious, systemic diseases as rheumatoid arthritis, metabolic disorders, malignancy and idiopathic [17]. CT is well accepted for evaluating pericarditis, although for assessment of constriction, echocardiography and MRI are more reliable. Findings include exudative or transudative, loculated fluid in the pericardial sac, in which differences in attenuation can be helpful. Thickening of the pericardial wall can be seen, and enhancement of the pericardial wall can be appreciated especially in active inflammatory pericarditis. Calcifications are seen in 50 percent of patients. Other findings include pleural effusions and ascites. It is of importance to recognize signs of constriction (Fig. 7).

### Pericardial fat necrosis

**Fig. 8:**
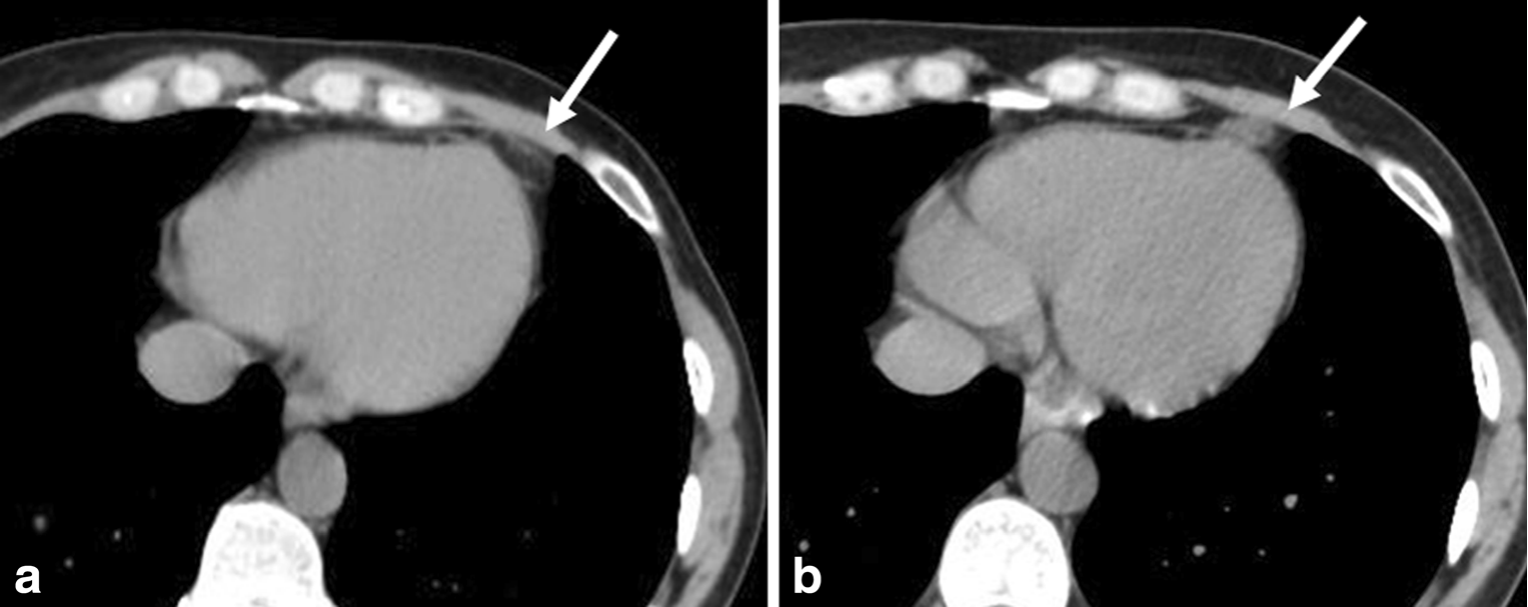
A 37-year-old female patient presented with acute non-radiating left chest pain. She had no cardiovascular risk factors. ECG showed sinus rhythm, and there were no ST or T wave abnormalities. Cardiac enzymes were normal, and the chest X-ray was unremarkable. Plain CT was performed for further evaluation. (**a**) Axial thoracic demonstrates a soft tissue lesion adjacent to the pericardium with a low attenuation coefficient (-39 HU). Based on the clinical and radiologic findings, the diagnosis of pericardial fat necrosis was suspected. (**b**) A month later, another axial thoracic CT showed that the lesion became smaller, with increased attenuation (15 HU)

Pericardial fat necrosis (PFN) is a recently recognized cause of severe chest pain [18]. In the acute phase, the appearance of the lesion is quite similar to the so-called epiploic appendagitis (epiploic appendage fat necrosis). Although the pathogenesis of PFN is not clear, in some cases lesions have shown to be attached to the heart by a pedicle; acute torsion has been postulated as the cause of necrosis, which leads to sharp pleuritic chest pain. The appearance of the PFN depends on the age of the lesion. In the early phases, the fat composition of the lesion is well depicted. Within days, the lesion becomes smaller and denser that represents the evolution into scar tissue. PFN is included in the diagnosis of cardiophrenic mass lesions that also consider primary and secondary fat-containing neoplasms, pericardial cyst, and diaphragmatic hernia. CT demonstrates an ovoid soft tissue mass in close relation to the pericardium with low attenuation and possible adjacent thickening of the pericardium. Given the unique appearance of PFN in the initial phase at thoracic CT, it is important for the radiologist to be familiar with its features, Fig. 8.

## Pulmonary

**Fig. 9:**
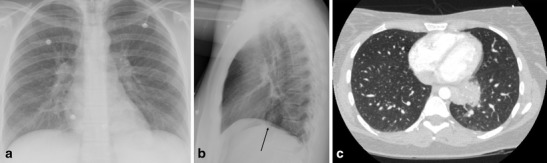
A 20-year-old old female presented with retrosternal chest pain, fever, and elevated white blood counts. (**a-b**) PA and lateral chest radiograph. Initially the plain radiograph was read as without abnormalities. (**c**) Axial chest CTA, performed under suspicion of a pulmonary embolism shows a consolidation in the left lower lobe, which can be seen as a subtle abnormality on the lateral chest x-ray in (**b**) (arrow)

A variety of pulmonary aetiologies can cause chest pain. Some of the common entities include pneumonia and pneumothorax, as well as pulmonary embolism. These entities typically do not cause a diagnostic challenge on routine chest CT examination, although they may be hard to interpret on plain radiographs, Fig. 9.

### Chronic thromboembolic pulmonary hypertension

Acute pulmonary embolism is a frequent diagnosis associated with acute chest pain. The incidence is 70 per 100,000 per year [19]. CT is a well-established method for diagnosing pulmonary embolism, as well as its acute complications, including right ventricular overload [20, 21]. Embolisms usually represent blood-thrombus, as a result from deep venous thrombosis, although incidentally fat- , tumour-, or air-embolisms are encountered.

**Fig. 10:**
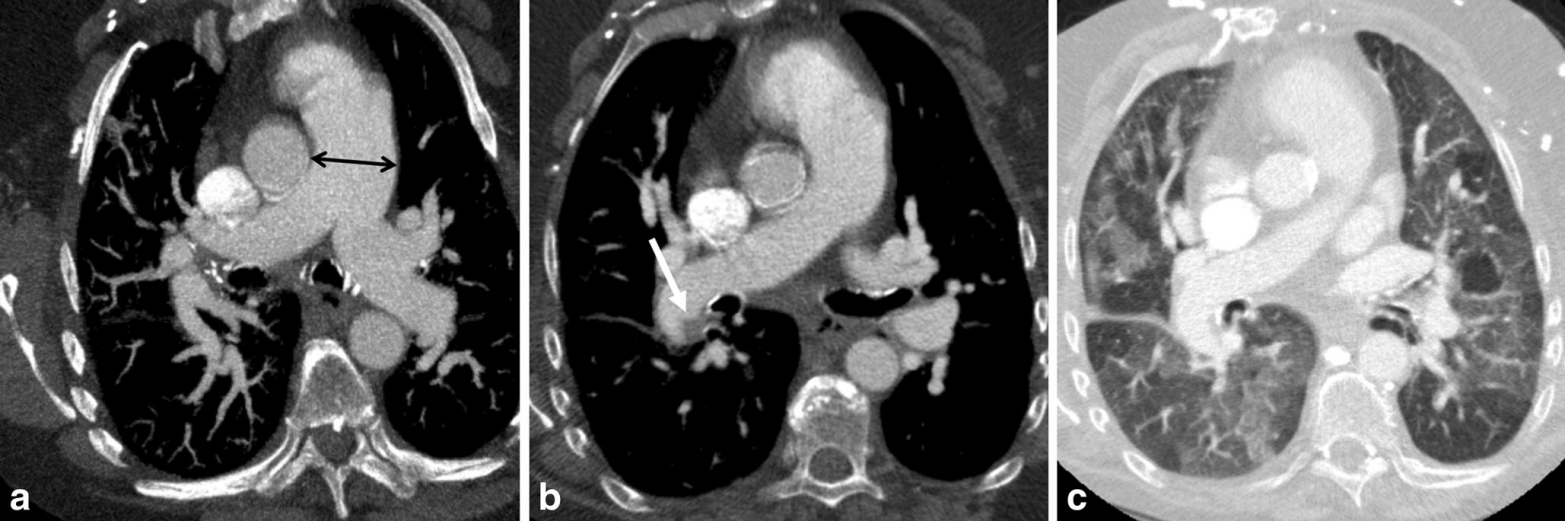
A 62-year-old male presented to the ED with exertional chest pain and progressive dyspnoea. He is known with systemic lupus erythematosus. (**a**) The CT images show a dilatation of the pulmonary trunk and the left and right pulmonary arteries (arrow), (**b**) with eccentric thrombotic material in the right pulmonary artery (arrow), and (**c**) mosaic attenuation consistent with chronic thromboembolic pulmonary hypertension

Chronic thromboembolic pulmonary hypertension (CTEPH) may occur as a complication of unresolved acute pulmonary embolism. The diagnosis is often delayed due to the atypical complaints of exertional chest pain, syncope, dyspnoea, and often leads to recurrent visits to the ED without a definite diagnoses. The severity of the presenting symptoms is determined by the hemodynamic changes, thus based on the load of thrombus and obstruction. The incidence of CTEPH is about 0.1-0.5 percent of patients who survive initial acute pulmonary embolism [22]. CT findings include those of arterial hypertension with mosaic attenuation. Moreover, additional signs include complete or partial obstruction of the pulmonary arteries, eccentric thrombus, calcified thrombus, bands, webs, and poststenotic dilatation. There are often signs of right ventricular overload (i.e., dilatation of the right ventricle, dilatation of inferior vena cava and hepatic veins). Pulmonary thromboendarterectomy is considered in symptomatic patients, Fig. 10.

### Pulmonary fat embolism

**Fig. 11:**
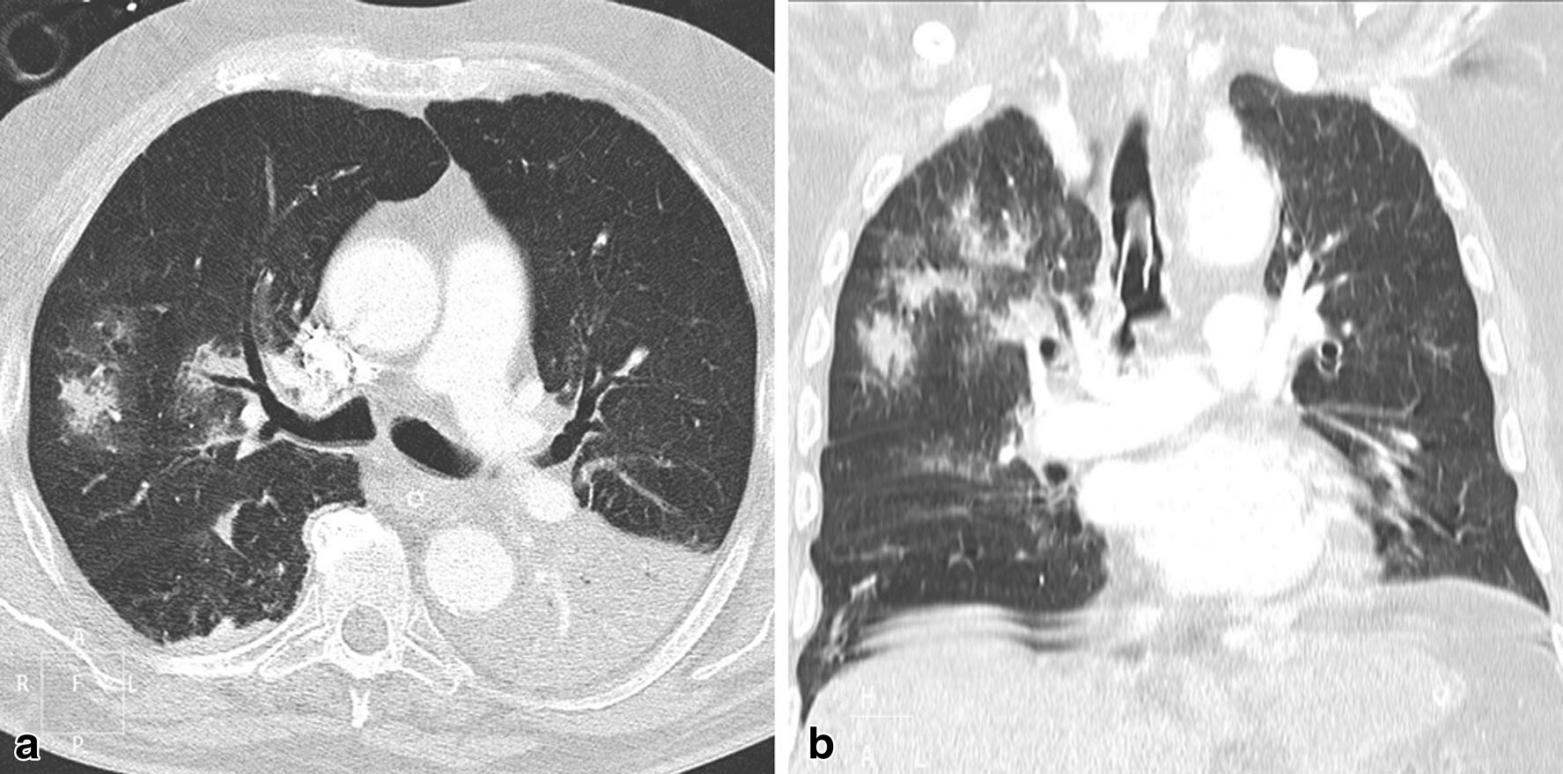
A 67-year-old female patient presented with new onset of chest pain and dyspnoea. The patient was diagnosed with an intertrochanteric femur fracture the day before and initial thoracic CT showed no abnormalities. Pulmonary CTA was ordered in order to rule out pulmonary embolism. (**a-b**) Thoracic CTA, (**a**) axial and (**b**) coronal, showed alveolar- and ground glass opacities, and nodules with halo sign distributed predominantly in the right upper lobe, and a left lower lobe consolidation. The nodular and ground glass pattern in the clinical context raised the diagnosis of pulmonary fat embolism

Fat embolism syndrome is usually associated with traumatic or iatrogenic conditions [23]. Although subclinical fat emboli are assumed to be present in virtually all patients with traumatic injuries, the clinical syndrome is reported to develop between 0.5 % and 2 % following fractures of the long bones. The radiological differential diagnosis includes lung contusion, pulmonary oedema, and aspiration. The association of bilateral ground glass opacities and nodular pattern in a recent traumatic context is highly suggestive of pulmonary fat embolism. Pathologic correlation is hard to find, but experimental studies show that these nodules and ground glass opacity probably represent areas of oedema, haemorrhage, and atelectasis, Fig. 11.

## Miscellaneous

### Intramural esophageal dissection

Intramural oesophageal dissection is a rare entity, associated with a mucosal tear, mostly developing during endoscopy or eating. The clinical presentation may greatly vary, although the diagnosis should be considered in the case of an acute onset of odynophagia during or after eating [24]. The dissection creates a false and true lumen and may be associated with a perforation leading to pneumopericardium, pneumomediastium, or leakage to the pleural space. Complete rupture of the oesophagus (Boerhaave syndrome) or a partial tear (Mallory Weiss-tear) are among other cases of oesophageal related chest pain, and are associated with forceful emesis.

**Fig. 12:**
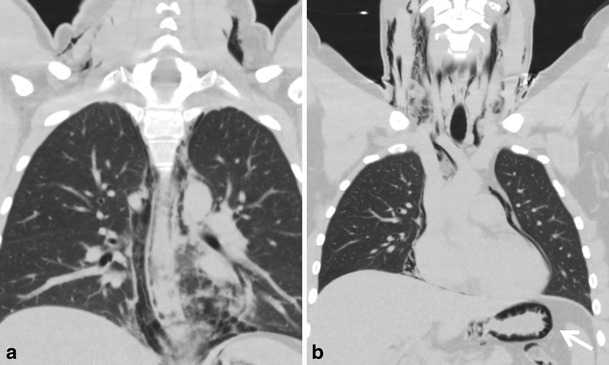
A 25-year-old male presented at the ED with complaints of chest pain, radiating to the shoulders. The pain started during evening dinner with an impacted food bolus. His history revealed dysphagia. At the ED he acutely developed dyspnoea after drinking water. Subcutaneous emphysema developed. A routine chest X-ray and thoracic CT examination were performed for further evaluation. (**a**) The coronal thoracic CT shows intramural gas extending from the level of the upper oesophagus to the (**b**) stomach (arrow) accompanied with extensive pneumopericardium, pneumomediastinum, and subcutaneous emphysema, due to perforated oesophageal dissection. At endoscopy, two foci of perforation in the distal oesophagus were found, which were successfully treated by placement of a (retrievable) covered stent

Oesophageal dissection can be treated conservatively (avoidance of oral intake). However, in cases of severe extension and an associated perforation, surgical intervention or endoscopic guided stenting should be considered, Fig. 12.

### Patent ductus arteriosus

**Fig. 13:**
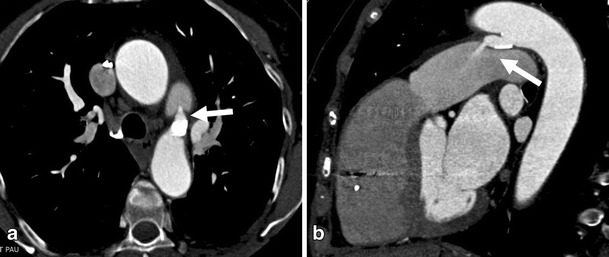
A 61-year-old female patient visited the ED with chest pain and mild dyspnoea. The patient was diagnosed with atrial fibrillation and had carried a pacemaker since 2000. Echocardiography showed a questionable patent ductus arteriosus (PDA). A thoracic CTA was ordered. Patent ductus arteriosus. (**a**) CT axial image shows a tubular structure (arrow) abutting aorta with adjacent coarse calcification. (**b**) Sagital multiplanar reconstruction shows enhanced blood flowing from aorta to pulmonary artery via patent ductus arteriosus

Patent ductus arteriosus (PDA) is a result of failure of the physiological mechanism that closes the fetal duct between the left pulmonary artery and the thoracic aorta after birth. Isolated PDA is estimated to account for 10-12 % of all congenital heart diseases [25]. Presentation in adult life is rare. Clinical manifestations of PDA mainly depend on ductus size and the pressure gradient that may lead to pulmonary hypertension and consequently to chest pain. PDA can be tolerated for many years without clinical signs or symptoms, but patients may become symptomatic when associated with acquired conditions such as recurrent pneumonia or the development of chronic obstructive pulmonary disease. For adults, transcatheter occlusion of the patent ductus is the preferred treatment. The CT provides valuable pre-treatment information regarding localization, size, and shape of the duct [26], Fig. 13.

## Technical considerations

Cardiac CT techniques are still rapidly evolving. The newest commercially available systems image the whole heart in a single breath-hold and tube rotation, limiting effective patient radiation dose.

Dual rule-out (ECG-gated) CT, tailored to rule out aortic dissection and pulmonary embolism, as well as triple rule-out CT, additionally for ruling out significant coronary artery disease, have shown their value in the diagnostic process, also by suggesting alternative diagnoses. In the appropriate setting using the appropriate scanning protocols, a triple rule out protocol may reduce time for patient triage, radiation dose, and costs [27, 28].

## Conclusion

The expected increase in thoracic, tailored rule-out CT examinations will urge the (general) radiologist to gain confidence in detecting and reporting alternative diagnoses on regular thoracic CT or CTA examinations requested in the setting of acute chest pain. Familiarity with some of the aortic, coronary, pericardial, and pulmonary diagnoses, and the sometimes subtle imaging findings will help to suggest alternative diagnoses, thereby avoiding diagnostic delay.
